# Advances in Endoplasmic Reticulum Stress Research—Insights from the Special Issue “Endoplasmic Reticulum Stress and Apoptosis”

**DOI:** 10.3390/ijms26062487

**Published:** 2025-03-11

**Authors:** Beáta Lizák, Orsolya Kapuy

**Affiliations:** Institute of Molecular Biology, Semmelweis University, H-1094 Budapest, Hungary; lizak.beata@semmelweis.hu

The endoplasmic reticulum (ER) is pivotal in maintaining the internal homeostasis of the cell. A common feature of various widespread diseases (e.g., diabetes and inflammatory bowel diseases) is the disturbance in the ER luminal microenvironment and thus the induction of ER stress in cells. Consequently, many studies have focused on understanding the exact mechanism of the maintenance of ER stability and the details of the ER stress response [1]. This Special Issue, entitled “Advances in Endoplasmic Reticulum Stress and Apoptosis” features seven papers presenting novel insights into the molecular biology of ER, regarding signalling pathways in various diseases, maintaining the ER lumen redox equilibrium, and potential ways to modify the ER stress response by naturally occurring molecules.

A review article written by Kapuy mainly focused on the mechanism of unfolded protein response (UPR) induced by the accumulation of misfolded proteins upon ER stress. The primary function of UPR is to minimise the extent of damage and restore the system to its original or a new homeostatic state by means of autophagy-dependent self-cannibalism. However, an excessive level of stress can result in apoptosis. This review systematically synthesised recent knowledge on the dynamic characteristics of UPR, drawing upon recent theoretical and molecular biological data. Additionally, Kapuy provided a detailed examination of the mechanism of action of the kinetic features of the positive and negative feedback loops within the molecular network that controls cellular decisions regarding life and death in response to ER stress.

There is also ongoing scientific research into the possible use of natural substances to delay the adverse effects of ER stress [2]. For example, natural rare sugars (such as D-allulose and D-fructose) are an alternative category of sweeteners with positive physiologic and metabolic effects both in in vitro and animal models. Cimino et al. has shown that D-allulose treatment in substitution for glucose was able to prevent, at various concentrations tested, palmitic acid (PA), which is a trigger for hypertrophic adipocytes. They confirmed that the D-allulose effects were associated with reduced C/EBP-β, PPARγ, *IL-6*, and *IL-8* expression, and it was also effective in reducing NF-κB nuclear translocation. In addition, D-allulose pretreatment reduced, in a dose-dependent way, ER stress markers (such as XBP1s, eIF2a-P, GRP98, and CHOP), restoring their levels to those of the control at the higher concentration tested. These data confirmed that these rare sugars can bear free radical scavenger activities and reduce ER stress. Their results suggest that these naturally occurring rare sugars could be a valuable alternative to sweeteners. For similar reasons, Holczer et al. investigated the active ingredient in broccoli, called sulforaphane (SFN), using systems biological methods. SFN has been shown to induce autophagy and apoptosis in a concentration- and time-dependent manner. Short SFN treatment at low concentrations has been found to promote autophagy, whereas longer treatment at higher concentrations was able to induce cell death. It has been also demonstrated that pre- or co-treatment with a low concentration of SFN combined with short and long ER stress was able to promote cell survival via autophagy induction in each treatment. Therefore, their results suggest the potential medical importance of SFN in ER stress-related diseases.

The ER lumen is considered to have a unique redox composition maintained by the selectively permeable ER membrane and the special redox active intraluminal enzymes [3]. This feature is indispensable to support the organelle functions in protein processing and biomolecule synthesis. The two main redox pairs in the ER lumen—glutathione (GSH)/glutathione disulphide (GSSG) and NADPH/NADP—are very different from their cytosolic situation. The GSH/GSSG is mostly oxidized, supporting oxidative protein folding, while the pyridine nucleotides are mostly reduced, sustaining reducing reactions. In the cytosol, the two systems are coupled by glutathione reductase and thioredoxin reductases, while in the ER lumen, these enzymes are absent. Veszelyi et al. provided extensive evidence to show the absence of thioredoxin (Trx) and thioredoxin reductase (TrxR) from the ER lumen, using enzyme activity measurements, co-localization experiments, and in silico analysis. Furthermore, they also observed how deteriorative it would be to couple the two redox systems in the ER lumen by overexpressing ER-targeted Trx and TrxR. The presence of these proteins significantly decreased the cell viability and triggered apoptosis. These data confirm the importance of the ER’s distinctive redox balance.

Aside from redox molecules, redox active enzymes are also part of the ER’s special redox equilibrium. Although cytosolic thioredoxin is not present in the lumen, many intraluminal enzymes bear the thioredoxin-like domain, including the protein disulphide isomerase (PDI) family members participating in thiol–disulfide exchange reactions [4]. These proteins partake in essential processes in the ER such as oxidative protein folding, ERAD, trafficking, calcium homeostasis, etc. More than 20 family members have been described so far, but the exact function of many remains unknown. Bidooki et al. studied TXNDC5, a PDI family member, whose function as a protective factor in various stress provoking conditions was described in previous publications. ER stress pathways are main factors in the background of non-alcoholic fatty liver disease. Bidooki et al. examined the role of TXNDC5 function in hepatic cells during ER malfunction. The effect of TXNDC5 knockout on ER stress pathways was studied in AML12 cells using three different stressors: thapsigargin, palmitic acid, and tunicamycin. Depending on the mechanism of stress, they observed differently involved signalling pathways altered by the absence of TXNDC5. TXNDC5 knockout also induced cellular ROS production and contributed to normal mitochondrial activity. These results provide detailed insights into the role of TXNDC5 protein in regulating cell homeostasis.

When studying ER stress, it is particularly important to study proteins that may be somehow protective for the cell. Fei et al. introduced Homer scaffold protein 1 (Homer1), as a postsynaptic scaffold protein, to study its impact on the ER stress-associated TXNIP/NLRP3-mediated cell pyroptosis after ischemic injury. Their analysis showed that the overexpression of Homer1 significantly increased the AMPK phosphorylation levels, suggesting that Homer1 can regulate ER stress-related TXNIP/NLRP3-mediated pyroptosis through the AMPK signalling pathway. However, it cannot be ruled out that the protective effects against retinal ischemia of Homer1 are achieved in a variety of ways. Therefore, this is worth exploring further in the future.

NF-κB transcription factor family is a master regulator of cellular signalling pathways, controlling a wide range of processes from inflammation to lymphocyte development. It is also involved in apoptosis and cellular survival, and contributes to several types of cancer [5]. Msweli et al. provided a comprehensive analysis of the evolution of NF-κB proteins and their distribution in different species, demonstrating important data to understand the development of the immune system.

Unravelling the molecular mechanism and regulatory context of the ER stress response mechanism provides an opportunity to understand the dynamics of the regulatory network. In addition, finding potential targets for therapeutic intervention in ER stress is of long-term importance for their medical purposes and is expected to receive increasing attention in the coming years.
